# Design and Analysis of Differential Compensated Eddy Current Displacement Sensors

**DOI:** 10.3390/s25123578

**Published:** 2025-06-06

**Authors:** Yuliang Bian, Kun Zhang, Tiehua Ma

**Affiliations:** 1 State Key Laboratory of Extreme Environment Optoelectronic Dynamic Measurement Technology and Instrument, School of Electrical and Control Engineering, North University of China, Taiyuan 030051, China; 2 Anhui Engineering Research Center for Intelligent Computing and Information Innovation, School of Computer and Information Engineering, Fuyang Normal University, Fuyang 236041, China

**Keywords:** differential, eddy current, displacement, large range

## Abstract

The traditional eddy current displacement sensor is limited by the diameter of the probe coil, and the measuring range is relatively small. In order to improve the range of an eddy current displacement sensor, a differential compensated eddy current displacement sensor (DCECDS) is proposed in this paper. The DCECDS is designed with three coils and is characterized by a large measuring range and good linearity. Based on the analysis of the working principle of DCECDS, the corresponding equivalent circuit model and mathematical model were established in this study. The effects of excitation frequency, inner diameter and thickness of excitation coil on the sensor performance were investigated. Based on the simulation results, the structural parameters of the sensor were designed and verified by experiments. The experimental results show that the measuring range of the DCECDS designed in this study can reach 1.76 times the diameter of the probe coil, which is 3.5 times higher than that of the traditional eddy current displacement sensor. The measuring range of the eddy current displacement sensor is effectively improved, which provides an important reference and practical value for the design of eddy current displacement sensors.

## 1. Introduction

Displacement measurement technology has a wide range of applications in the industrial field. Through displacement measurement, movement, vibration and size in the industrial production process can be monitored in real time, providing important data support for industrial automation control [1,2,3].

The eddy current displacement sensor is a non-contact measurement sensor based on the principle of electromagnetic induction. It has the advantages of a wide measurement range, fast response speed, high sensitivity and strong anti-interference ability. These advantages make eddy current displacement sensors widely used in many fields, such as industry, electronics and electric power [4,5,6].

However, the measurement range of traditional eddy current sensors on the market is generally about 0.5 times the diameter of the probe coil, which generally has the disadvantage of a small measurement range, which greatly limits its application range [7]. Therefore, the research on how to improve the range of eddy current displacement sensors has important practical significance, and many researchers have performed extensive relevant research.

Some researchers improve the performance of sensors by optimizing the sensors’ input signals. In [8], the influence of sine signal, triangle signal and pulse signal on the transducer was compared, and they concluded that the linearity of triangle signal is better than that of sine signal, and the sensitivity of pulse signal is better than that of sine signal. In [9], by establishing the circuit simulation model of the double-coil inductive displacement sensor, the influence law of the excitation signal frequency on the linearity and sensitivity of the sensor was studied, and the determination method of the optimal frequency obtained. In [10], a mathematical model was established to study the influence of different coil excitation modes on the working performance of the displacement sensor, and it was concluded that the linearity of the dual-coil series three-wire (DCSTW) was the best, which was 35.67% higher than the dual-coil parallel differential (DCPD) and 17.52% higher than the dual-coil series four-wire (DCSFW). By establishing finite element simulation models, some researchers studied the influence of various probe parameters on the output performance of eddy current displacement sensors and on this basis improved the performance of eddy current displacement sensors [11,12]. But these studies could not effectively improve the range of sensors.

Other researchers have improved sensor performance by improving the sensor processing circuitry. In [13], an improved eddy current sensor drive circuit was proposed, which used a crystal oscillator circuit with power boost to replace the traditional colpitts oscillator circuit, so that the eddy current sensor had good linearity (1.12%) and sensitivity (2.14 V/mm). In [14], the resonant impedance inversion method based on linearization of transfer curve adopted a negative impedance converter at the sensor input to compensate for the displacement probe loss, extending the linear range of the sensor from 0.25 mm to 3.75 mm, which is about 44% of the diameter of the 8 mm probe. In [15], a vortex displacement sensor using Coriolis circuit was proposed, which extends the measurement range of the sensor to 56% of the probe diameter by measuring Rp (resonant loss resistance) and optimizing the probe sensitivity. Although the above research can improve the range of the sensor, the effect is very limited.

In practical applications, it is necessary to extend the range of eddy current displacement sensors as far as possible under the premise of ensuring the linearity of eddy current displacement sensors. This paper presents a design scheme of an eddy current displacement sensor with differential compensation structure, which greatly improves the range and linearity of the sensor. Taking the structure of the differential compensated coil as the research object, this study describes the working principle of the differential compensated eddy current displacement sensor (DCECDS) in detail, establishes the equivalent circuit model and finite element simulation model of the DCECDS, and analyzes the law of the influence of different coil parameters on the sensor’s detection range and linearity. The optimal design results of the probe coil parameters of the DCECDS are given. Finally, the feasibility and correctness of the design are verified by experiments, which provides important reference value for the design of eddy current displacement sensors.

## 2. Theoretical Basis

### 2.1. Structural Design of DCECDS

**Figure 1:**
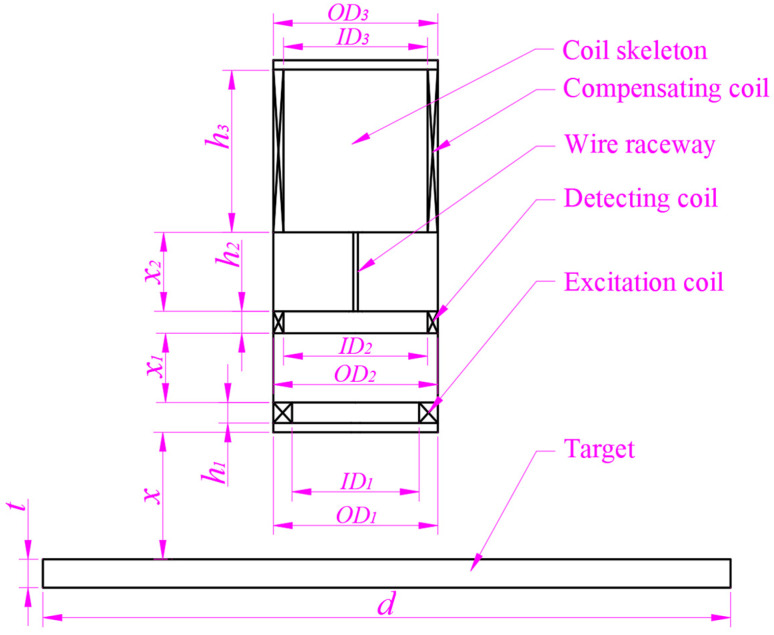
DCECDS probe structure.

The probe structure of the DCECDS is mainly composed of three coils, a skeleton, an electrical interface and a shell. The three coils include the excitation coil, the detection coil and the compensation coil. The internal coil structure of the probe of the DCECDS is shown in Figure 1 below. The high frequency excitation signal is loaded into the excitation coil and used to generate an induced magnetic field. The detection coil and the compensation coil are connected in reverse series, and the series output end of the two coils is the output end of the sensor signal. Among them, the detection coil is mainly used to sense the changes in magnetic field intensity caused by the displacement changes between the probe and the object being measured. The compensation coil is mainly used to correct the zero-point potential of the output signal of the detection coil and improve the linearity of the output signal.

### 2.2. Mathematical Model of DCECDS

#### 2.2.1. Principle of Eddy Current Testing

**Figure 2:**
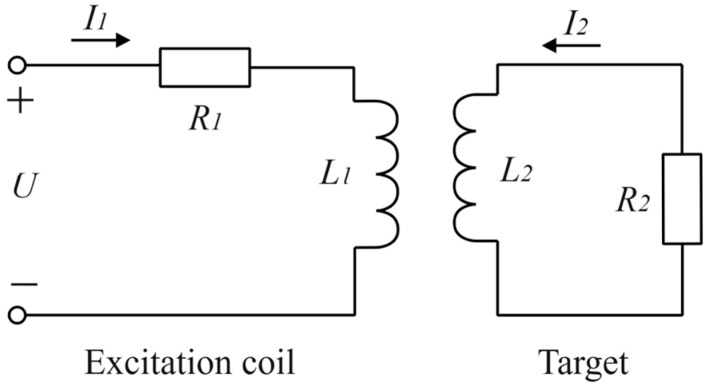
Equivalent circuit diagram for eddy current testing.

As shown in Figure 1, when a high-frequency alternating current is loaded in the excitation coil inside the sensor probe, the alternating current will generate an electromagnetic field around the probe. Because the coil itself has a certain resistance value, it consumes electrical energy and generates Joule heat. Therefore, the excitation coil can be regarded as a primary circuit in series with an inductor and a resistor. The alternating electromagnetic field generates eddy currents in the measured conductor target, which can be regarded as a secondary loop composed of inductors and resistors in series. When the output end of the sensor is open, the influence of the measured target on the detection coil and the compensation coil can be ignored. When the parasitic capacitance of the coil is ignored, the coupling relationship between them can be explained by the equivalent transformer model, and the equivalent circuit is shown in Figure 2 [16].

The interaction between the excitation coil inside the sensor and the measured target can be described by the following mathematical model. According to Kirchhoff’s voltage law, the voltage relationship between the primary and secondary circuits can be expressed as:

(1) $$\left\{\begin{array}{c} R_{1}I_{1}+j\omega L_{1}I_{1}-j\omega MI_{2}=U\\ R_{2}I_{2}+j\omega L_{2}I_{2}-j\omega MI_{1}=0 \end{array}\right.$$

In the above formula, $R_{1}$ and $L_{1}$ are respectively the resistance and inductance of the probe excitation coil, $R_{2}$ and $L_{2}$ are respectively the equivalent resistance and inductance of the measured conductor target, ω is the excitation signal frequency, and M is the mutual inductance coefficient between the inductive probe and the measured target, which is related to the distance between the two. After simplifying the above formula, the equivalent current in the excitation coil and the measured target after the coupling of the two is obtained as follows:

(2) $$\left\{\begin{array}{c} I_{1}=\frac{U}{R_{1}+\frac{\omega^{2}M^{2}R_{2}}{R_{2}^{2}+\omega^{2}L_{2}^{2}}+j\omega \left(L_{1}-\frac{\omega^{2}M^{2}L_{2}}{R_{2}^{2}+\omega^{2}L_{2}^{2}}\right)}\\ I_{2}=\frac{M\omega^{2}L_{2}+j\omega MR_{2}}{R_{2}^{2}+\omega^{2}L_{2}^{2}}I_{1} \end{array}\right.$$

When the probe excitation coil is close to the measured metal surface, the equivalent impedance of the excitation coil is expressed as follows:

(3) $$Z=\frac{U}{I_{1}}=R_{1}+\frac{\omega^{2}M^{2}R_{2}}{R_{2}^{2}+\omega^{2}L_{2}^{2}}+j\omega \left(L_{1}-\frac{\omega^{2}M^{2}L_{2}}{R_{2}^{2}+\omega^{2}L_{2}^{2}}\right)$$

Therefore, the equivalent resistance $R^{\prime }$ and the equivalent inductance $L^{\prime }$ of the probe excitation coil can be expressed as follows:

(4) $$\left\{\begin{array}{c} \begin{array}{c} \begin{array}{c} R^{\prime }=R_{1}+\frac{\omega^{2}M^{2}R_{2}}{R_{2}^{2}+\omega^{2}L_{2}^{2}}\\ L^{\prime }=L_{1}-\frac{\omega^{2}M^{2}L_{2}}{R_{2}^{2}+\omega^{2}L_{2}^{2}} \end{array} \end{array} \end{array}\right.$$

As can be seen from Formula (4), when there is a measured target, the resistance of the induction coil increases while the inductance decreases. The change of resistance and inductance is related to the frequency of the excitation signal and the distance between the coil and the measured target. It can be seen that the resistivity ρ and permeability μ of the measured metal conductor, the distance x between the excitation coil and the measured target, and the angular frequency ω of the excitation signal will all affect the impedance Z of the excitation coil through eddy and magnetic effects. The relationship between them can be expressed as $Z=F\left(x,\mu ,\rho ,\omega \right)$; if only the displacement x changes in the above parameters while the rest of the parameters remain unchanged, the impedance Z becomes a single value function of the displacement parameter, so that the size of the displacement can be determined according to the change in impedance.

#### 2.2.2. Mathematical Model of DCECDS

**Figure 3:**
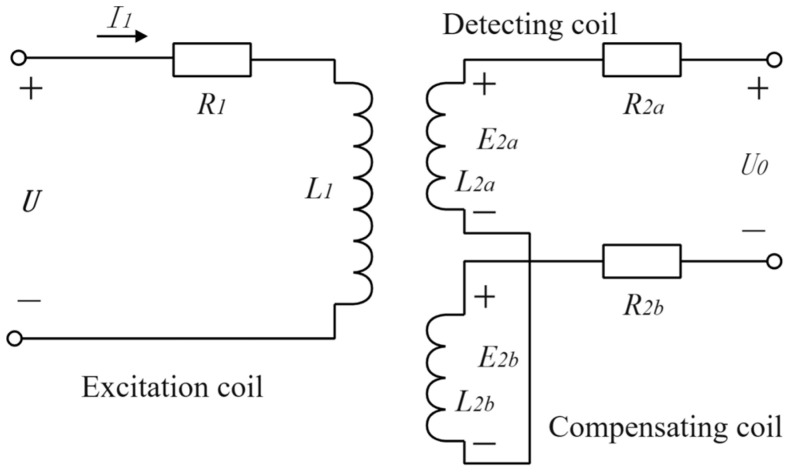
Equivalent circuit model of DCECDS.

The main function of the detection coil and the compensation coil in the probe of the DCECDS is to measure the impedance change of the excitation coil and thus determine the displacement x. The detection coil and the compensation coil in the sensor probe are connected in series in reverse. Under ideal conditions ignoring iron loss, magnetic resistance of the magnetic conductor and distributed capacitance of the coil, the equivalent circuit is shown in Figure 3 below.

When the excitation voltage U is loaded in the excitation coil, according to the working principle of the transformer, the induced electromotive force $E_{2a}$ and $E_{2b}$ will be generated in the detection coil and the compensation coil, respectively.

When there is no detection target in the detection range and the output is open,

(5) $$I_{1}=\frac{U}{R_{1}+j\omega L_{1}}$$

According to the law of electromagnetic induction, the expression of the induced potential in the detection coil and the compensation coil is as follows:

(6) $$E_{2a}=-j\omega M_{1}I_{1}$$

(7) $$E_{2b}=-j\omega M_{2}I_{1}$$

In the above formula, $M_{1}$ is the mutual inductance between the excitation coil and the detection coil, and $M_{2}$ is the mutual inductance between the excitation coil and the compensation coil.

Since the detection coil and the compensation coil are connected together in reverse series, the total output $U_{0}=E_{2a}-E_{2b}$. When the output terminal is in an open circuit state, it can be obtained from the above relationship:

(8) $$U_{0}=E_{2a}-E_{2b}=-\frac{j\omega \left(M_{1}-M_{2}\right)U}{R_{1}+j\omega L_{1}}$$

The effective values of the output voltage are as follows:

(9) $$U_{0}=\frac{\omega \left(M_{1}-M_{2}\right)U}{\sqrt{R_{1}^{2}+{\left(\omega L_{1}\right)}^{2}}}$$

#### 2.2.3. DCECDS Output Zeroing Principle

According to Formulas 6 and 7, the sizes of $E_{2a}$ and $E_{2b}$ are related to the mutual inductance of the excitation coil, the detection coil and the compensation coil, and the mutual inductance between the two coils is only related to the geometry, size, number of turns and the relative position between the coils but not to the current.

When there is no measured target, the mutual inductance coefficient $M_{1}$ between the excitation coil and the detection coil and the mutual inductance coefficient $M_{2}$ between the excitation coil and the compensation coil can be controlled by adjusting the number of turns of the compensation coil so that $E_{2a}=E_{2b}$. By performing the preceding operations, we can adjust the zero output of the sensor and reduce the zero residual voltage so that the output voltage of the sensor $U_{0}=0$. In this way, the output signal can be zeroed when making the sensor probe, and the sensitivity and range of the sensor output signal can be increased.

When there is a detection target in the sensor detection range, $M_{1}\neq M_{2}$,$\;\Delta M=M_{1}-M_{2}$. The effective value of the output voltage is as follows:

(10) $$U_{0}=\frac{\omega \left(M_{1}-M_{2}\right)U}{\sqrt{R_{1}^{2}+{\left(\omega L_{1}\right)}^{2}}}$$

When there is a measured target in the detection range, due to the influence of eddy current effect, the DC resistance $R_{1}$ and inductance $L_{1}$ of the excitation coil, and the mutual inductance coefficient $M_{1}$ and $M_{2}$ between the excitation coil, the detection coil and the compensation coil will be changed. Because the distances between the excitation coil, the detection coil and the compensation coil are different, the influence on the mutual inductance coefficient $M_{1}$ and $M_{2}$ is also different. As can be seen from Formula (10), the distance between the probe coil and the measured target can be converted into the change of the output voltage, that is, the output voltage is a function of the distance between the sensor and the measured target. The displacement between the probe and conductor can be calculated by obtaining the induced voltage output of the sensor through the subsequent signal processing circuit.

## 3. Multi-Coil Finite Element Model of Sensor Probe

### 3.1. The Geometric Structure of Simulation Model

**Figure 4:**
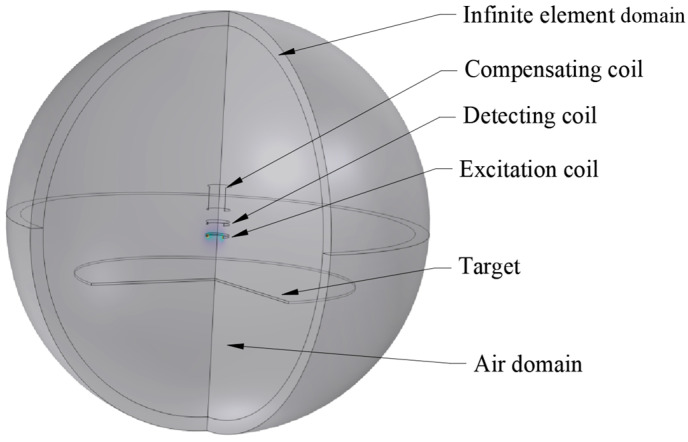
Finite element model of DCECDS.

The following uses the AC/DC function of COMSOL Multiphysics 6.1 software to establish a probe multi-coil simulation model and conduct a simulation study on the output results of the sensor with multiple design parameters in the model [17]. The influence law of different coil parameters on the range and linearity of sensor output signal is explored, and the optimal parameter value of sensor probe coil size is finally determined. This study focuses on the influence of frequency f=ω/2π, inner diameter $D_{1}$ and height h on the range and linearity of the output signal when the outer diameter $D_{2}$ of the excitation coil is fixed. The simulation model effect of the probe coil of the DCECDS established in COMSOL software is shown in Figure 4.

### 3.2. Parameter Design of Simulation Model

The low-frequency electromagnetic field of AC/DC module in COMSOL software is selected for simulation. Three “uniform multiturn coils” are arranged in the magnetic field, corresponding to the excitation coil, the detection coil and the compensation coil, respectively. In order to simulate the actual application environment and improve the accuracy of the simulation results, the infinite element domain is set outside the air domain. Copper is chosen as the coil material and aluminum alloy as the measured target. The main design parameters of the differential compensation eddy current displacement sensor probe are shown in Table 1 below.

### 3.3. Grid Division

**Figure 5:**
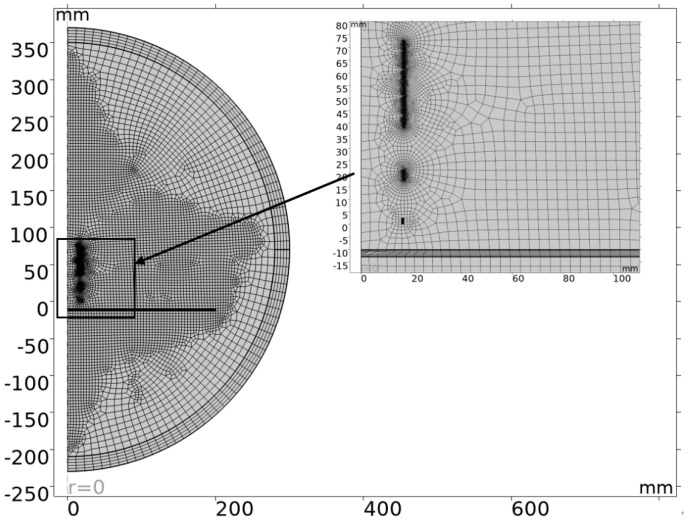
Grid diagram.

The refinement of the mesh division in the simulation model determines the accuracy and convergence speed of the simulation. In order to improve the accuracy of simulation results, the grid sequence type is a user-controlled grid, and the mesh cell size is ultra-refined, with the minimum cell size being 0.01 mm. The model uses a free quadrilateral grid, and six boundary layers are added inside the grid of coils and measured conductors. The meshing results are shown in Figure 5.

## 4. Analysis of Simulation Results

### 4.1. Influence of Different Excitation Frequencies on Output Results

**Figure 6:**
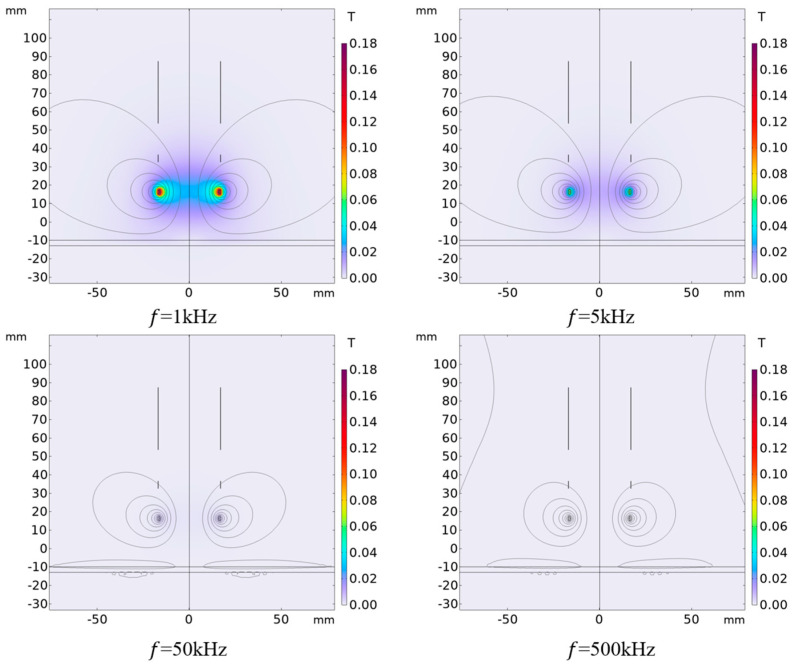
Distribution of magnetic induction intensity with different excitation frequencies ($ID_{1}=32\;\mathrm{mm},\;h_{1}=2.5\;\mathrm{mm},\;x=25\;\mathrm{mm}.\;$The other parameters of the coil are set as per Table 1).

**Figure 7:**
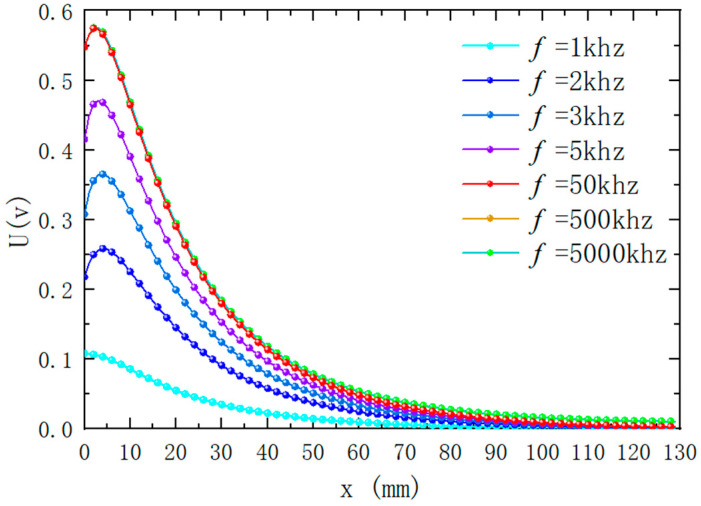
Variation of output voltage with distance when excitation frequency is different ($ID_{1}=32\;\mathrm{mm},\;h_{1}=2.5\;\mathrm{mm},\;x=25\;\mathrm{mm}.\;$The other parameters of the coil are set as per Table 1).

When only the signal frequency f loaded in the excitation coil is changed, the simulation results of the magnetic induction intensity around the differential compensation eddy current displacement sensor are shown in Figure 6. It can be seen from the figure that under different excitation frequencies, the magnetic induction intensity distribution is basically the same, but the intensity is different. Figure 7 shows the change of sensor output voltage with different lifting distances when the excitation frequency is different. The following can be seen from the figure: (1) The lower the frequency of the excitation signal, the smaller the sensitivity of the sensor output signal in the entire detection range; the higher the frequency of the excitation signal, the higher the sensitivity of the sensor output signal in the entire detection range. (2) When the frequency of the excitation signal exceeds 50 kHz, the frequency of the excitation signal continues to increase, the sensitivity of the sensor output signal basically does not change within the range of 40 mm, and the sensitivity becomes lower beyond the range of 30 mm. (3) When the excitation frequency exceeds 1 kHz, in the detection range of 0 to 5 mm, the output signal of the sensor increases with the increase in the detection distance, and when the detection range of 5 mm is exceeded, the output signal of the sensor decreases with the increase in the detection distance. According to the above analysis results, a higher measuring range and sensitivity can be obtained by choosing 50 kHz as the frequency of the excitation signal in practical application.

### 4.2. Influence of Different Excitation Coil Inner Diameters on Output Results

**Figure 8:**
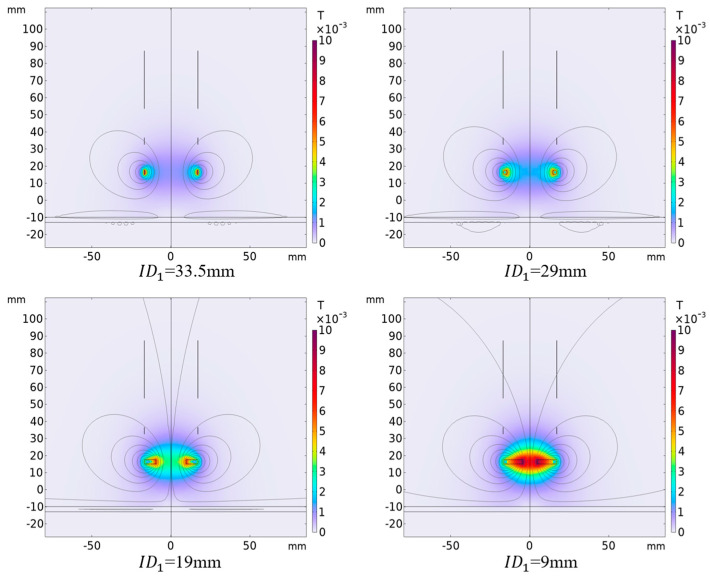
Distribution of magnetic induction intensity with different inner diameters of excitation coils ($\;h_{1}=2.5\;\mathrm{mm},\;f=50\;\mathrm{kHz},\;x=25\;\mathrm{mm}.\;$The other parameters of the coil are set as per Table 1).

**Figure 9:**
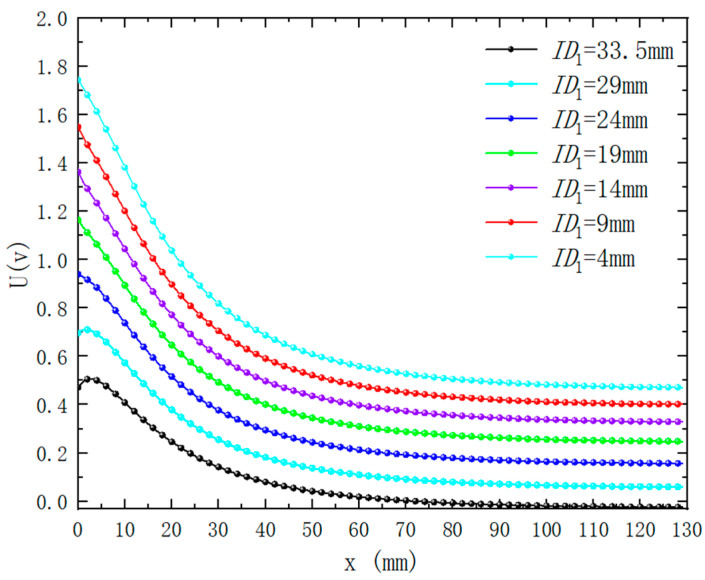
The variation of output voltage with distance when the inner diameter of excitation coil is different ($\;h_{1}=2.5\;\mathrm{mm},\;f=50\;\mathrm{kHz},\;x=25\;\mathrm{mm}.\;$The other parameters of the coil are set as per Table 1).

Under the premise that the outer diameter of the excitation coil is 34 mm and the height is 2.5 mm, the inner diameter of the excitation coil is controlled, and the number of turns of the excitation coil will also change accordingly. When the inner diameter of the exciting coil is different, the magnetic induction intensity distribution around the differential compensation eddy current displacement sensor is simulated as shown in Figure 8. It can be seen from the figure that the magnetic induction intensity and distribution around the probe change significantly when the inner diameter of the exciting coil is different. Figure 9 shows the change of sensor output voltage with different lifting distances when the inner diameter of the excitation coil is different. As can be seen from the figure, with the reduction in the inner diameter of the excitation coil, that is, the increase in the number of turns of the coil, the sensitivity of the sensor output signal also increases. At the same time, with the reduction in the inner diameter of the sensor excitation coil, the nonlinear relationship between the output signal and the distance becomes linear. However, when the inner diameter of the coil is too small, that is, the number of turns of the coil is too large, it will make the linearity of the output signal deteriorate during close detection. Therefore, in practical application, the inner diameter of the excitation coil is selected to be 9 mm, which can obtain higher sensitivity and better linearity at close range.

### 4.3. Influence of Excitation Coil Height on Output Result

**Figure 10:**
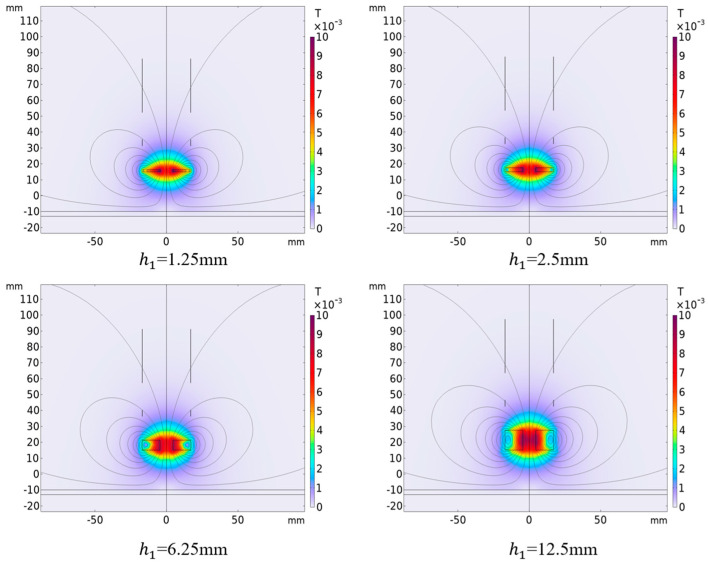
Magnetic induction intensity distribution with different excitation coil heights ($ID_{1}=9\;\mathrm{mm},\;f=50\;\mathrm{kHz},\;x=25\;\mathrm{mm}.\;$The other parameters of the coil are set as per Table 1).

**Figure 11:**
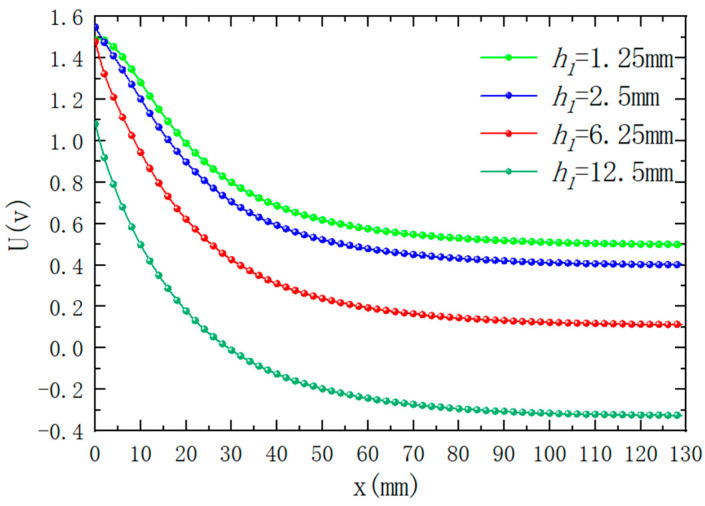
When the height of the excitation coil is different, the output voltage changes with the lifting distance ($ID_{1}=9\;\mathrm{mm},\;f=50\;\mathrm{kHz},\;x=25\;\mathrm{mm}.\;$The other parameters of the coil are set as per Table 1).

Under the premise of controlling the inner diameter of the excitation coil to be 9 mm, when only the height of the excitation coil is changed, the magnetic induction intensity distribution simulation results around the differential compensation eddy current displacement sensor. The results are shown in Figure 10. It can be seen from the figure that the magnetic induction intensity and distribution around the probe change significantly when the excitation coil height is different. Figure 11 shows the variation of sensor output voltage with different lifting distances when the excitation coil height is different. As can be seen from the figure, the height of the excitation coil has a great impact on the output signal. Among them, when the height of the excitation coil is smaller, the sensitivity of the output signal will become smaller, and the height is too small; in the detection range of a short distance, the output signal and the detection distance will have nonlinear characteristics. When the height of the excitation coil increases, the sensitivity of the output signal will increase, but when the height is too large, the output signal will change from forward to reverse (*h* = 12.5 mm), which is because the induced voltage generated in the compensation coil is greater than the induced voltage generated by the detection coil, which will increase the difficulty of the design of the subsequent signal processing circuit. Therefore, the height of the excitation coil is selected to be 6.25 mm in practical application, which not only ensures the sensitivity of the sensor but also simplifies the signal processing circuit.

Through the above analysis, the final design parameters of the excitation coil of the differential compensation eddy current displacement sensor are shown in Table 2 below.

## 5. Experiment and Analysis

**Figure 12:**
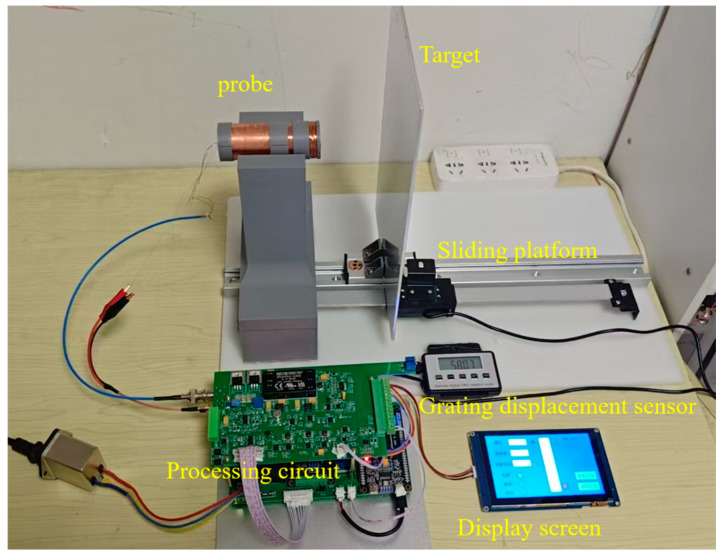
Experimental platform.

The internal coil of the probe of the DCECDS was fabricated according to the parameter data in Table 1 and Table 2. The experimental platform built is shown in Figure 12. The experimental platform consists of a probe, the measured target, a moving slider, a display screen, a grating displacement sensor and a processing circuit. The aluminum plate selected for the measured target is 300 (mm) × 200 (mm) × 3 (mm). High-precision grating displacement sensor is used to display the actual distance between the probe and the measured target. The main functions of the signal processing circuit are first, to generate an excitation signal with a frequency of 50 kHz; second, to appropriately amplify, filter and detect the output signal of the probe, converting it into a DC voltage signal corresponding to the displacement, and then using an analog-to-digital converter to convert it into the corresponding digital quantity for display on the screen.

**Figure 13:**
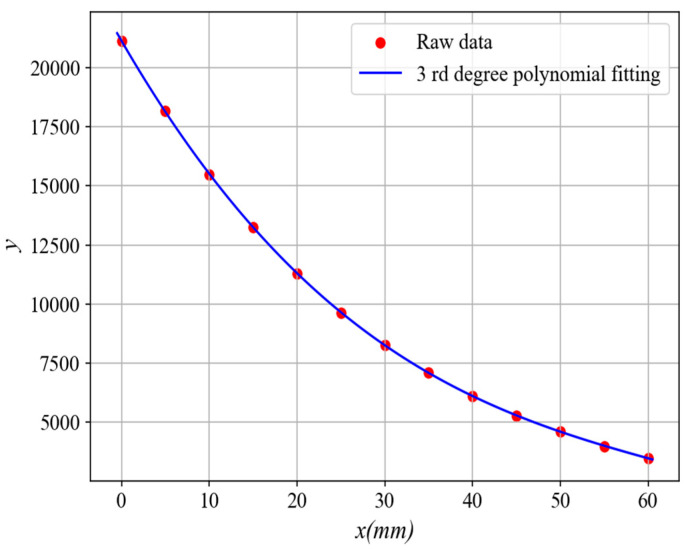
Sensor calibration curve.

**Figure 14:**
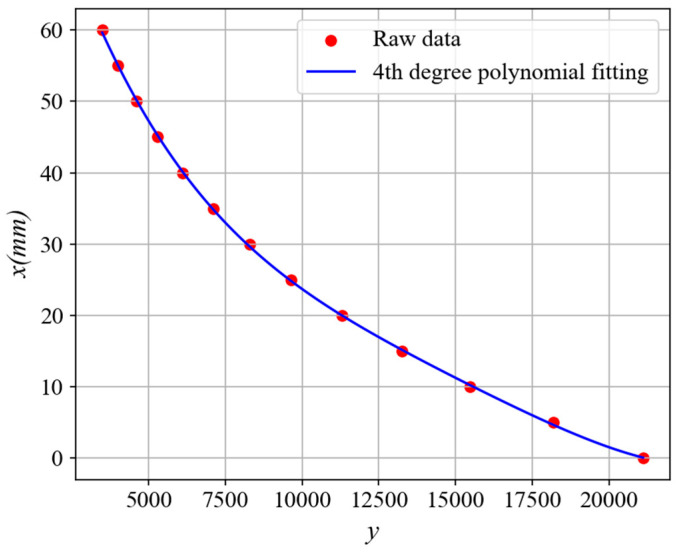
Inverse function of sensor calibration curve.

By controlling the guide rail to realize the movement of the measured target, the experimental platform is first used to calibrate the sensor, and the original data are shown in Figure 13. The horizontal coordinate *x* represents the distance from the probe to the target being measured, and the vertical coordinate *y* represents the digital quantity output by the analog-to-digital converter corresponding to *x*. As can be seen from the figure, the actual output result of the sensor output is basically consistent with the simulation result, which further verifies the correctness of the sensor design. The calibration curve equation obtained by fitting the original data with a third degree polynomial is $y=-0.04309x^{3}+8.378x^{2}-642x+21140$, and then fitting the inverse function of the calibration curve with a fourth degree polynomial is shown in Figure 14. The corresponding curve equation is $x=9.682\times 10^{-16}y^{4}-5.955\times 10^{-11}y^{3}+1.403\times 10^{-6}y^{2}-0.017y+104.6$. Finally, the sensor output signal is linearized, and the linear equation of the sensor output is U=−0.75x+4.5, where *x* is the measured distance, and *U* is the equivalent voltage value of the sensor after data processing.

**Figure 15:**
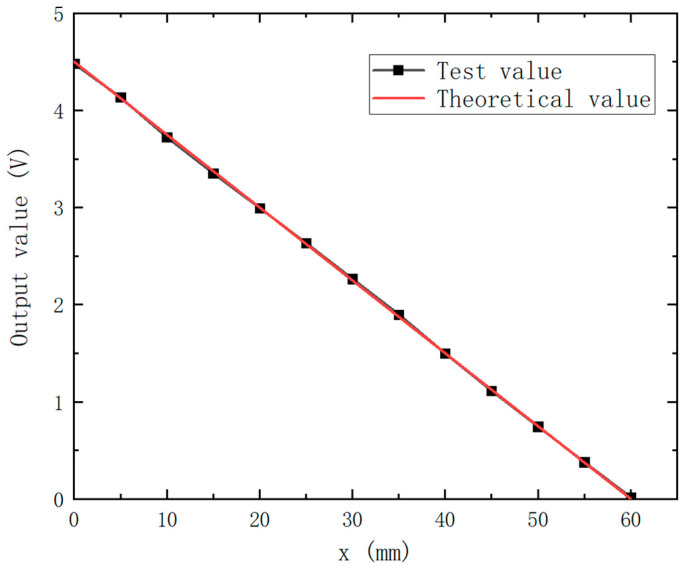
Sensor test results.

In the measuring range of 0–60 mm, 13 points are selected every 5 mm to test the sensor. The test results are shown in Figure 15, which shows that the test data are basically distributed around the line y=−0.75x+4.5. According to the calculation, the maximum measurement error of the sensor is only 0.58%, and the measurement sensitivity is 75 mv/mm in the measuring range of 60 mm. The experimental results show that this research can effectively extend the range of eddy current displacement, so that the effective range of the sensor can reach 1.76 times the diameter of the probe coil, which is about 3.5 times higher than that of the conventional eddy current displacement sensor.

## 6. Conclusions

In this study, a new type of eddy current sensor is designed, which adopts a differential compensation three-coil structure. The following conclusions are drawn through simulation and experimental analysis.

1. Improving the frequency of the excitation signal can effectively increase the sensitivity of the sensor output signal, but when the frequency of the excitation signal increases to more than 50 kHz, the sensitivity of the sensor output signal is basically unchanged in the near detection, and the sensitivity is slightly reduced in the remote detection.
2. Under the premise that the outer diameter and height of the control coil are unchanged, the inner diameter of the excitation coil becomes smaller, and the sensitivity of the sensor output signal becomes higher. The inner diameter of the coil can be controlled to improve the linearity of the output signal when the sensor is measured at close distance.
3. The eddy current sensor designed with differential compensation coil structure can reach the target detection range of about 1.76 times the outer diameter of the excitation coil, which is about 3.5 times higher than that of the conventional eddy current displacement sensor.

The above research shows that the eddy current displacement sensor with this structure can effectively expand its range, which provides important reference value for the research of large-range eddy current displacement sensors.

## Figures and Tables

**Table 1:** Main parameters of the original structure size of the probe.

Argument	Value	Unit	Explanation
$OD_{1}$	34	mm	outside diameter of excitation coil
$ID_{1}$	<34	mm	inner diameter of excitation coil
$h_{1}$	>0	mm	height of excitation coil
$x_{1}$	15	mm	coil spacing
*d*	200	mm	target diameter
*t*	3	mm	target thickness
*f*	>1	kHz	excitation frequency
*x*	>0	mm	detection distance
$OD_{2}$	34	mm	outside diameter of detecting coil
$ID_{2}$	33.9	mm	inner diameter of detecting coil
$h_{2}$	4.7	mm	height of detecting coil
$OD_{3}$	34	mm	outside diameter of compensating coil
$ID_{3}$	33.9	mm	inner diameter of compensating coil
$h_{3}$	35	mm	height of compensating coil
$x_{2}$	17	mm	coil spacing

**Table 2:** The main parameters of the probe after optimization.

Argument	Value	Unit	Explanation
$OD_{1}$	34	mm	outside diameter of excitation coil
$ID_{1}$	9	mm	inner diameter of excitation coil
$h_{1}$	6.25	mm	height of excitation coil
*f*	50	kHz	excitation frequency

## Data Availability

The data presented in this study are available on request from the corresponding author.
